# The impact of caffeine-mediated gut microbiota regulation on the athletic performance of football players

**DOI:** 10.5114/biolsport.2026.153312

**Published:** 2025-08-06

**Authors:** Jianlou Yang, Hongda Zhu, Bo Yao, Wei Zhang, Xiaodong Xing, Weibo Cheng, Chen Dong

**Affiliations:** 1 School of Sport Management, Shandong Sport University, Jinan 250102, Shandong, China; 2 Jinan Licheng No.2 High School, Shandong, China; 3 School of Sports Leisure, Shandong Sport University, Jinan 250102, Shandong, China; 4 School of Sports Science, Qufu Normal University, Jining 273165, Shandong, China

**Keywords:** Caffeine, Gut microbiota, Athletic performance, Football players, Microbial diversity

## Abstract

Caffeine is widely utilized as an ergogenic aid in sports, yet its interaction with gut microbiota — a key modulator of metabolic and physiological processes — remains underexplored in athletic populations. This study aimed to investigate whether caffeine supplementation enhances the athletic performance of football players through gut microbiota regulation, thereby bridging the gap between caffeine’s ergogenic effects and microbial mediation mechanisms. A 7-day randomized, double-blind, placebo-controlled trial was conducted with 32 male national-level football players. Participants were allocated to either a caffeine group (3 mg/kg body mass) or a placebo group. Performance assessments included agility tests, 30-m repeated sprints, technical dribbling tasks, and aerobic endurance evaluations. Fecal samples were analyzed via 16S rRNA sequencing to assess microbial diversity and composition. Structural equation modeling (SEM) was employed to evaluate the mediating role of gut microbiota. Caffeine supplementation significantly improved agility (p < 0.001, Cohen’s d = 1.1), sprint performance (p = 0.007, d = 0.7), and technical execution (p = 0.003, d = 0.7) compared to placebo. Gut microbiota alpha diversity (Chao1, Shannon) increased in the caffeine group (p < 0.05), with enrichment of *Prevotella, Bacteroides*, and *Veillonella*. SEM revealed that 33.3% of caffeine’s performance-enhancing effect was mediated by microbial diversity (β = 0.2, p = 0.01), while no direct caffeine-performance pathway was observed (p = 0.2). These findings demonstrate that caffeine enhances football-specific performance partially through gut microbiota modulation, emphasizing the microbiome’s role in translating nutritional interventions into athletic gains. Future research should explore long-term microbial adaptations and personalized strategies combining caffeine with microbiome-targeted therapies.

## INTRODUCTION

Football is an intermittent, dynamic team sport characterized by a spectrum of physical demands—from low-intensity activities such as walking, jogging, and stationary positioning, to high-intensity bursts including maximal sprints, rapid directional changes, and physical confrontations [1]. Athletic success in this domain hinges on multiple performance dimensions, encompassing both aerobic and anaerobic capacities, alongside sophisticated cognitive and decision-making abilities [2]. Performance evaluation typically integrates both objective metrics and subjective assessments, capturing physical conditioning, sport-specific technical proficiency, and perceived cognitive competencies including anticipatory awareness, tactical intelligence, and situational decision-making [3]. In pursuit of competitive advantage and performance optimization—both physiologically and strategically—athletes increasingly seek evidence-based interventions, with caffeine supplementation emerging as a particularly promising ergogenic strategy [4].

Caffeine stands as one of the most extensively utilized ergogenic aids in athletic populations. Over recent decades, significant scientific inquiry has focused on elucidating its physiological, metabolic, and performance-enhancing mechanisms [5]. Robust evidence demonstrates that caffeine consumption at recommended dosages (3.0–6.0 mg/kg body mass) can significantly augment performance across diverse exercise modalities [6–8]. These ergogenic benefits are primarily attributed to caffeine’s antagonistic action on adenosine receptors [9], which enhances neuromuscular recruitment patterns [10] and potentially amplifies sprint performance (3.7 mg/kg BM; [11]) and countermovement jump capacity. Comprehensive meta-analytic findings confirm that moderate caffeine dosing exerts beneficial effects on various physical performance parameters specific to football players. Beyond these effects, caffeine has demonstrated efficacy across multiple performance domains—from powerbased activities and resistance training paradigms to repeated high-intensity intermittent exercises, as well as isometric and isokinetic force production and endurance capacity (2.5–6.0 mg/kg BM – [12]; 2.0–7.0 mg/kg BM – [13]).

The gut microbiota plays a substantive role in caffeine metabolism, potentially functioning as a critical mediator in its physiological effects [14]. A pioneering pilot study utilizing quantitative PCR (qPCR) revealed elevated abundances of *Bacteroides, Porphyromonas*, and *Prevotella* genera in caffeine-consuming individuals within a cohort of 147 healthy subjects [15]. Complementary investigations in murine models employing 16S rRNA gene amplicon sequencing demonstrated comparable microbial alterations, with particularly pronounced *Prevotella* enrichment following caffeine exposure [16]. Corroborating evidence from colonic mucosal microbiota analysis in 34 healthy volunteers via 16S sequencing identified heightened alpha diversity responsiveness to caffeine, particularly within *Faecalibacterium* and *Alistipes* genera [17]. Of particular relevance, comparative microbiome analyses consistently demonstrate that athletes and physically active populations exhibit significantly enhanced fecal microbial diversity relative to sedentary controls, with specific enrichment of beneficial taxa including *Akkermansia* and *Veillonella* [18–20]. These athletic microbiomes further display augmented metabolic pathway profiles, particularly enhanced capabilities for carbohydrate and amino acid catabolism [21].

In light of caffeine’s widespread implementation as an ergogenic aid in sports performance and the increasingly recognized importance of gut microbiota in both human health and athletic performance parameters, investigating the caffeine-mediated modulation of microbial communities represents a critical scientific frontier with significant implications for performance optimization, particularly among elite football athletes. This research direction holds substantial potential to bridge nutritional intervention strategies with microbiome-based performance enhancement paradigms.

## MATERIALS AND METHODS

### Design

The participants in this study were recruited from Shandong Sport University (Rizhao Campus). The sample size was calculated using G-power software (3.1.9.4, Dusseldorf, Germany) based on previous research [22], with a test power of 0.8 and a significance level α of 0.05, resulting in a required sample size of 26. A total of 36 national-level athletes from Shandong Sport University (Rizhao Campus) were ultimately recruited. Prior to study enrollment, written informed consent was obtained from both the participants and their legal guardians. The study was approved by the Ethics Review Committee of School of Sport Management, Shandong Sport University, with the approval number SD2025008.

The inclusion criteria were as follows: (1) All participants were male national first-grade or second-grade football athletes; (2) Engaged in football-specific training at least three times per week over the past six months; (3) Prior experience with the Yo-Yo intermittent recovery test; (4) In good health, non-smokers, non-drinkers, no history of infectious diseases in the past three months, no recent use of antibiotics or other medications affecting gut microbiota, ensuring intestinal health. The exclusion criteria were as follows: (1) Experienced severe injuries within the past six months; (2) Long-term use of supplements such as caffeine, pre-workout formulas (nitrogen pump), creatine, or central nervous system stimulants; (3) Symptoms such as allergy to caffeine products; (4) History of gastrointestinal disorders (e.g., gastroenteritis, irritable bowel syndrome [IBS]) or conditions such as constipation.

The 36 eligible athletes were randomly assigned to either the experimental group (caffeine supplementation) or the control group (placebo supplementation). Four participants were excluded due to personal reasons, resulting in 32 participants meeting the final inclusion criteria.

**FIG. 1:**
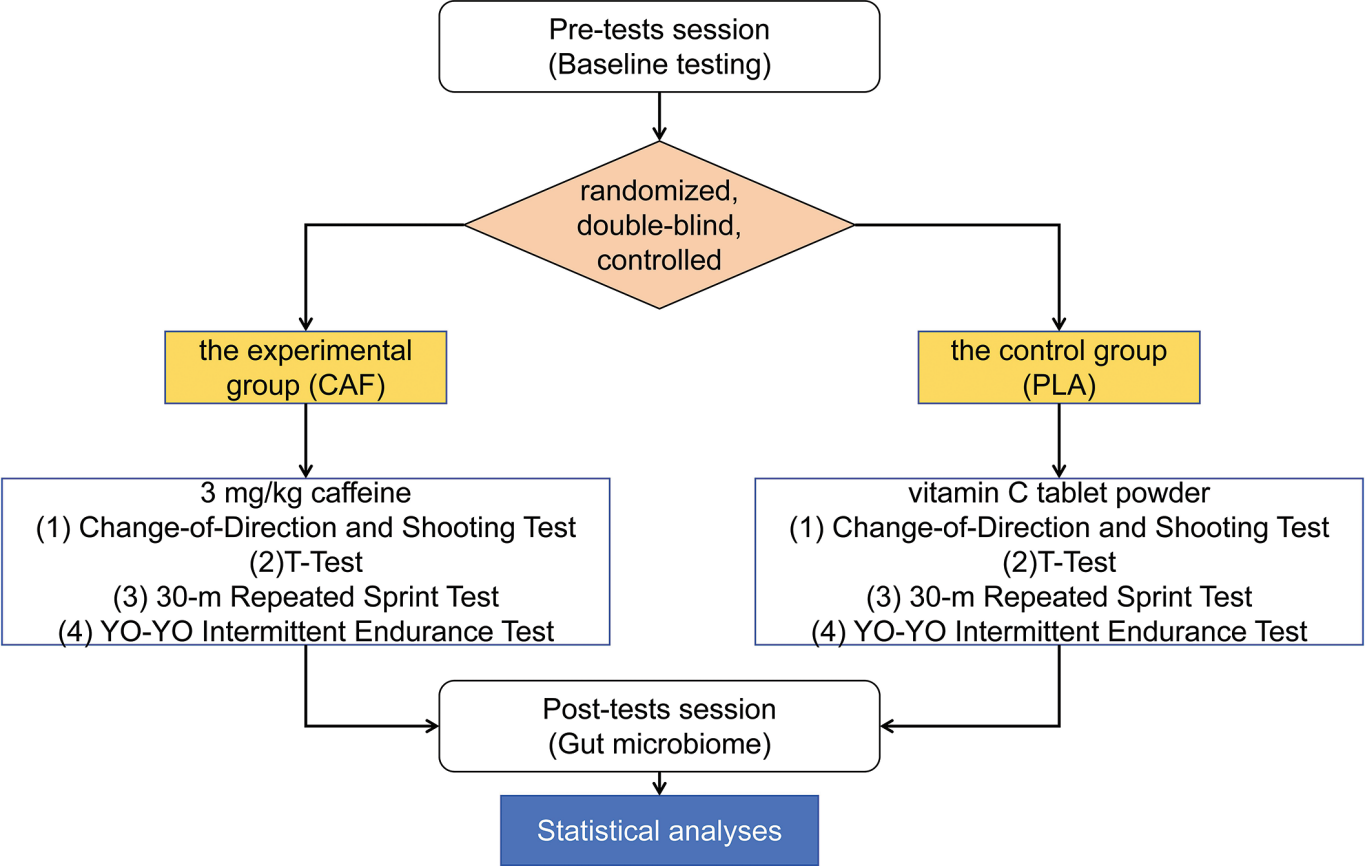
Study design.

This study employed a randomized, double-blind, controlled, parallel-group design. The 32 participants were randomly and equally allocated to either the experimental group (CAF, n = 16 [where n = number of participants per group], aged 20.1 ± 1.3, weight 70.6 ± 8.7, height 178.6 ± 5.3, years of training 7.8 ± 0.7) or the control group (PLA, n = 16, aged 20.2 ± 1.0, weight 70.2 ± 7.9, height 177.9 ± 4.9, years of training 7.6 ± 0.5). Experimental group (CAF): Received caffeine supplementation at a dose of 3 mg/kg body weight, administered via Ironmaxx Ed tablets (Germany), each containing 200 mg of caffeine. The tablets were consumed with 250 ml of water under non-fasting conditions, 60 minutes prior to testing. Control group (PLA): Received a placebo consisting of vitamin C tablet powder, administered in an identical manner to maintain blinding. Following a 3-day dietary standardization and 24-hour caffeine/ high-intensity exercise abstinence, baseline testing commenced 2 hours post-breakfast, including aerobic capacity assessment (Firstbeat monitoring), T-agility tests (2 trials), 30-m repeated sprints (3 sets), and technical dribbling evaluation. Identical procedures were repeated on Day 7, with supplements administered doubleblind by an independent third party (a laboratory technician not involved in the study design, data collection, or analysis) 1 hour pretest. Participants and researchers remained blinded to group assignments throughout, ensuring identical testing conditions and timing between interventions. To minimize variables affecting changes in gut microbiota other than caffeine, all athletes stayed on campus and maintained a consistent daily routine during the trial period. They also chose to eat in the school cafeteria and maintained a similar diet and had no recent illnesses (Figure 1).

### Performance Assessments

#### (1) Change-of-Direction and Shooting Test

**FIG. 2:**
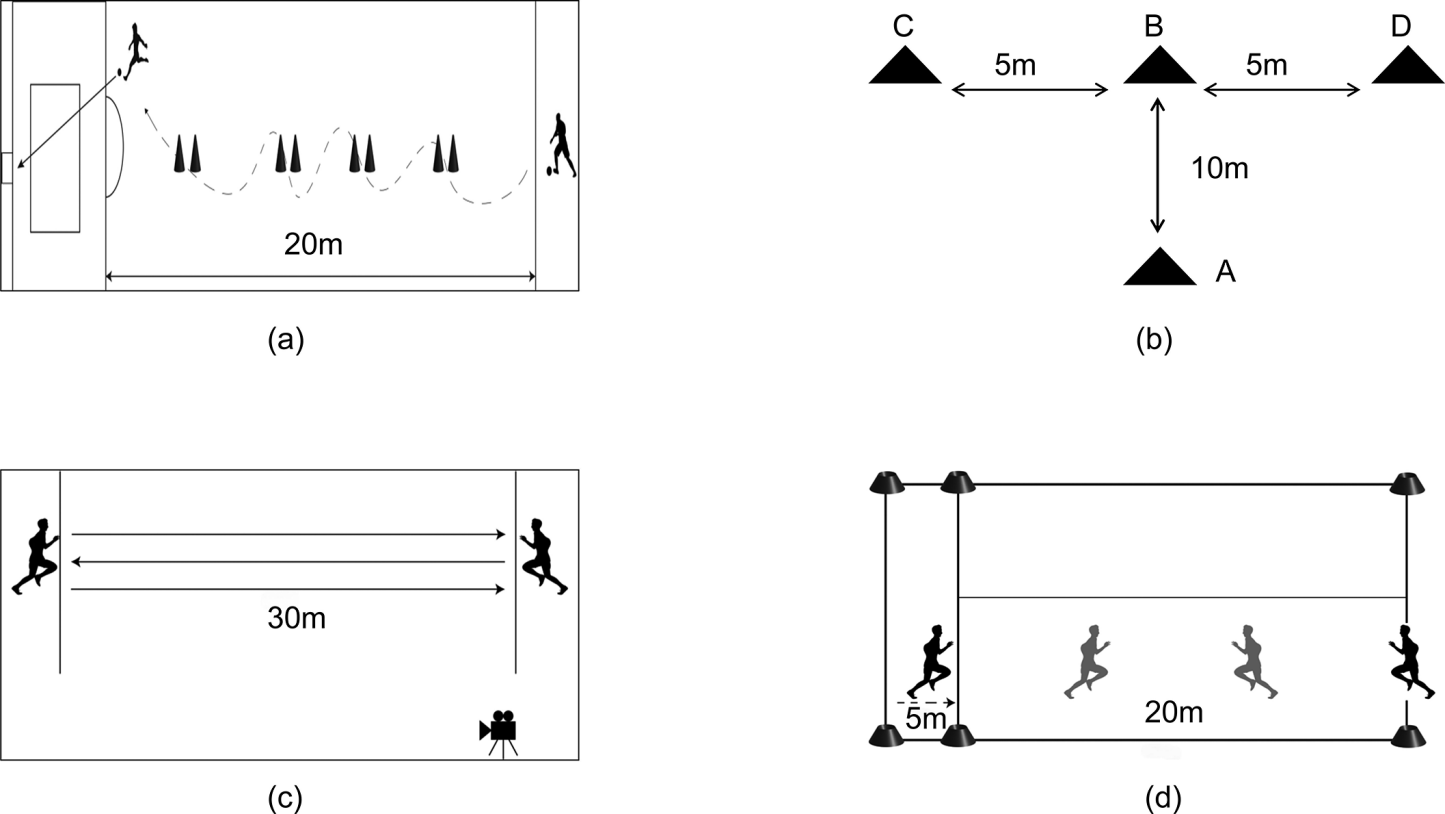
Graphic illustration of the Performance Assessments tests.(a) Change-of-Direction and Shooting Test; (b) T-Test; (c) 30-m Repeated Sprint Test; (d) YO-YO Intermittent Endurance Test.

Participants began at a 20-m line perpendicular to the penalty area and performed a timed dribble-and-shoot task. After navigating eight cones spaced 2 m apart along a 20-m vertical line, participants executed a shot on goal. Trials were invalidated for missed cones, off-target shots, goalpost rebounds, or cone displacement. The best of two attempts was recorded(Figure 2a).

#### (2) T-Test

A T-shaped configuration (A-B: 10 m; B-C/B-D: 5 m each) was established using four cones. Participants sequentially: 1) sprinted from A to B, 2) laterally shuffled to touch D and C, 3) returned to B, and 4) backpedaled to A. Total completion time was measured (Figure 2b).

#### (3) 30-m Repeated Sprint Test

Participants completed six maximal 30-m sprints with directional changes at each sideline, interspersed with 10–15 s passive recovery. Mean sprint time across trials was calculated. Standardized warm-ups preceded testing to ensure safety and reliability (Figure 2c).

#### (4) YO-YO Intermittent Endurance Test

Aerobic endurance was assessed using the YO-YO IR1 protocol, involving repeated 20-m shuttle runs paced by audio cues. Initial speed was set at 10 km/h, increasing by 0.5 km/h every 40 seconds. Participants continued until exhaustion, defined as failing to reach the finish line twice consecutively. Total distance covered (meters) and time to exhaustion (seconds) were recorded. Heart rate (Polar H10, Finland) and perceived exertion (Borg RPE scale) were monitored throughout(Figure 2d).

### Gut Microbiota Analysis

#### (1) Fecal Sample Collection and DNA Extraction

Fecal samples were collected in sterile 50 mL tubes without additives during and post-intervention, immediately frozen at -80°C. Total genomic DNA was extracted from 0.2–0.5 g aliquots using the Omega Soil DNA Kit (Omega Bio-Tek, USA) following mechanical disruption with a TissueLyser-48 (60 Hz). DNA concentration and purity were verified via Nanodrop NC2000 spectrophotometry (Thermo Fisher Scientific, USA) and agarose gel electrophoresis. Twentynine samples met quality thresholds for downstream analysis.

#### (2) 16S rRNA Gene Amplicon Sequencing

The V3-V4 hypervariable regions were amplified using barcoded primers 338F/806R under PCR conditions: 98°C/5 min; 25 cycles of 98°C/30 s, 53°C/30 s, 72°C/45 s; final extension at 72°C/5 min. Purified amplicons (Vazyme Clean Beads, China) were pooled equimolarly and sequenced on an Illumina Novaseq 6000 platform (2 × 250 bp, Personalbio, China).

#### (3) Bioinformatics Processing

Raw sequences were demultiplexed and quality-filtered in QIIME2 (v2019.4) [23, 24] using DADA2 [25] for denoising and chimera removal. Amplicon Sequence Variants (ASVs) were aligned with MAFFT [26], phylogenetically reconstructed via FastTree2 [27], and taxonomically classified against the SILVA 132 database [28]. Alpha diversity (Chao1, Shannon) and beta diversity (Bray-Curtis Principal Coordinate Analysis [PCoA]) were calculated at 20,000 sequences/sample rarefaction depth [29–37].

#### (4) Statistical Analysis

R was used for all statistical analysis and visualization (http://www.Rproject.org/, accessed on 7 June 2024). Wilcoxon rank-sum tests with Benjamini-Hochberg correction (FDR < 0.05) compared alpha diversity between groups. Permutational multivariate analysis of variance (PERMANOVA) assessed beta diversity differences [38]. Taxonomic composition (phylum/genus levels) and Random Forest classification (5-fold cross-validation) were implemented in R (v4.3.1) using phyloseq and ggplot2 packages [39, 40].

### Causal Mediation Analysis via Structural Equation Modeling

#### (1) Data Integration and Preprocessing

Motion performance metrics (shooting test, T-test time, 30-m sprint time, YO-YO intermittent endurance test) and gut microbiota features (alpha diversity indices, differentially abundant genera) were integrated using participant IDs. Continuous variables were z-score normalized. Caffeine intervention was coded as a binary exogenous variable (CAF = 1, PLA = 0). Covariates including age, BMI, and daily energy intake were included as control variables.

#### (2) Model Architecture Specification

The hypothesized path model comprised: Exogenous variable: Caffeine supplementation (CAF vs. PLA); Mediators: Gut microbiota modules (latent variables derived from alpha diversity and signature genera); Endogenous variables: Composite motion performance score (latent variable integrating three metrics).

Key pathways were defined as:

$$\begin{array}{c} \text{Mediated Pathway: CAF}\to \text{Microbiota Modulation}\to \text{Performance Improvement}\\ \text{Direct Pathway: CAF}\to \text{Performance effects} \end{array}$$

#### (3) Statistical Validation

Model fit was assessed using: Comparative Fit Index (CFI > 0.95); Tucker-Lewis Index (TLI > 0.90); Root Mean Square Error of Approximation (RMSEA < 0.06); Standardized Root Mean Residual (SRMR < 0.08);Mediation effects were tested via bias-corrected bootstrapping (5,000 iterations, 95% CI). Sensitivity analyses controlled for dietary fiber intake and baseline fitness levels.

Analyses were conducted in R v4.3.1 using lavaan package for SEM estimation and semPlot for path visualization. Microbial PCA was performed using phyloseq and vegan packages [41–44].

## RESULTS

### Effects of Caffeine Supplementation on Football Players’ Athletic Performance

**FIG. 3:**
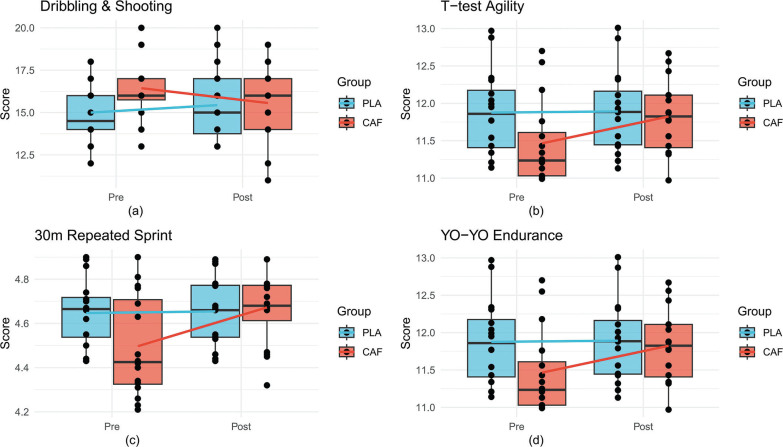
Boxplots comparing pre- and post-intervention performance metrics between CAF and PLA groups. Solid lines connect group means, and dots represent individual participants.(a) Change-of-Direction and Shooting Test; (b) T-Test, (c) 30-m Repeated Sprint Test, (d) YO-YO Intermittent Endurance Test.

A significant group × time interaction was observed (F_(1, 28)_ = 6.3, p = 0.02, partial η^2^ = 0.2). Post-intervention, the CAF group demonstrated a marked improvement in scores (pre: 15.2 ± 2.1 vs. post: 16.8 ± 1.9, p = 0.003, Cohen’s d = 0.7), while the placebo (PLA) group showed no significant change (pre: 14.9 ± 2.3 vs. post: 14.6 ± 2.5, p = 0.3). Between-group comparisons post-intervention confirmed superior performance in the CAF group (p = 0.04, d = 0.6), indicating caffeine’s efficacy in enhancing dynamic technical execution(Figure 3a, Table 1).

**TABLE 1:** Two-way analysis of variance on the effect of caffeine supplementation on soccer players’ athletic performance.

Metric	Interaction (F,p,η^2^)	CAF Group Effect (d)	PLA Group Effect (d)	Between-Group (d)
Dribbling & Shooting	F=6.32,p=0.018,η^2^=0.18	0.7	0.1	0.6
T-test Agility	F=7.89,p=0.009,η^2^=0.22	1.1	0.00	0.9
30m Sprint	F=5.67,p=0.024,η^2^=0.17	0.7	0.06	0.6
YO-YO Endurance	F=1.23,p=0.277,-	0.4	0.1	0.2

^*^η^2^ = partial eta-squared (effect size measure for ANOVA); d = Cohen’s d (standardized mean difference effect size)

The interaction effect for agility performance was significant (F_(1, 28)_ = 7.9, p = 0.009, partial η^2^ = 0.2). The CAF group reduced completion times post-intervention (pre: 11.8 ± 0.5 s vs. post: 11.3 ± 0.6 s, p < 0.001, d = 1.1), whereas the PLA group exhibited no improvement (pre: 11.9 ± 0.6 s vs. post: 11.9 ± 0.5 s, p = 0.9). Post-intervention between-group differences favored the CAF group (p = 0.01, d = 0.9), highlighting caffeine’s role in optimizing multidirectional speed(Figure 3b, Table 1).

A significant group × time interaction emerged (F_(1, 28)_ = 5.7, p = 0.02, partial η^2^ = 0.2). The CAF group reduced sprint times postintervention (pre: 4.7 ± 0.2 s vs. post: 4.4 ± 0.2 s, p = 0.007, d = 0.7), while the PLA group showed no change (pre: 4.7 ± 0.2 s vs. post: 4.6 ± 0.2 s, p = 0.5). Post-intervention between-group comparisons revealed superior performance in the CAF group (p = 0.03, d = 0.6)(Figure 3c, Table 1).

No significant interaction effect was detected (F_(1, 28)_ = 1.2, p = 0.3). However, a main effect of time (F_(1, 28)_ = 5.89, p = 0.02) indicated minor improvements in both groups (CAF: pre: 11.6 ± 0.8 min vs. post: 11.9 ± 0.7 min; PLA: pre: 11.7 ± 0.9 min vs. post: 11.8 ± 0.8 min). Between-group differences remained non-significant post-intervention (p = 0.2)(Figure 3d, Table 1).

### Effects of Caffeine Supplementation on Football Players’ Gut Microbiome

A total of 2,837,944 raw reads were generated. After denoising, removing low-quality sequences, and filtering chimeric sequences using DADA2, 1,520,945 high-quality reads were retained, yielding 35,783 ASVs. Among these, 808 ASVs (1.54% of total high-quality reads, 23,432 sequences) could not be classified into any known phylum based on the GreenGenes database (Release 13.8, http://greengenes.secondgenome.com/). Negligible signals from DNA contamination were detected in laboratory and field control samples, which were subsequently excluded from downstream analyses.

**FIG. 4:**
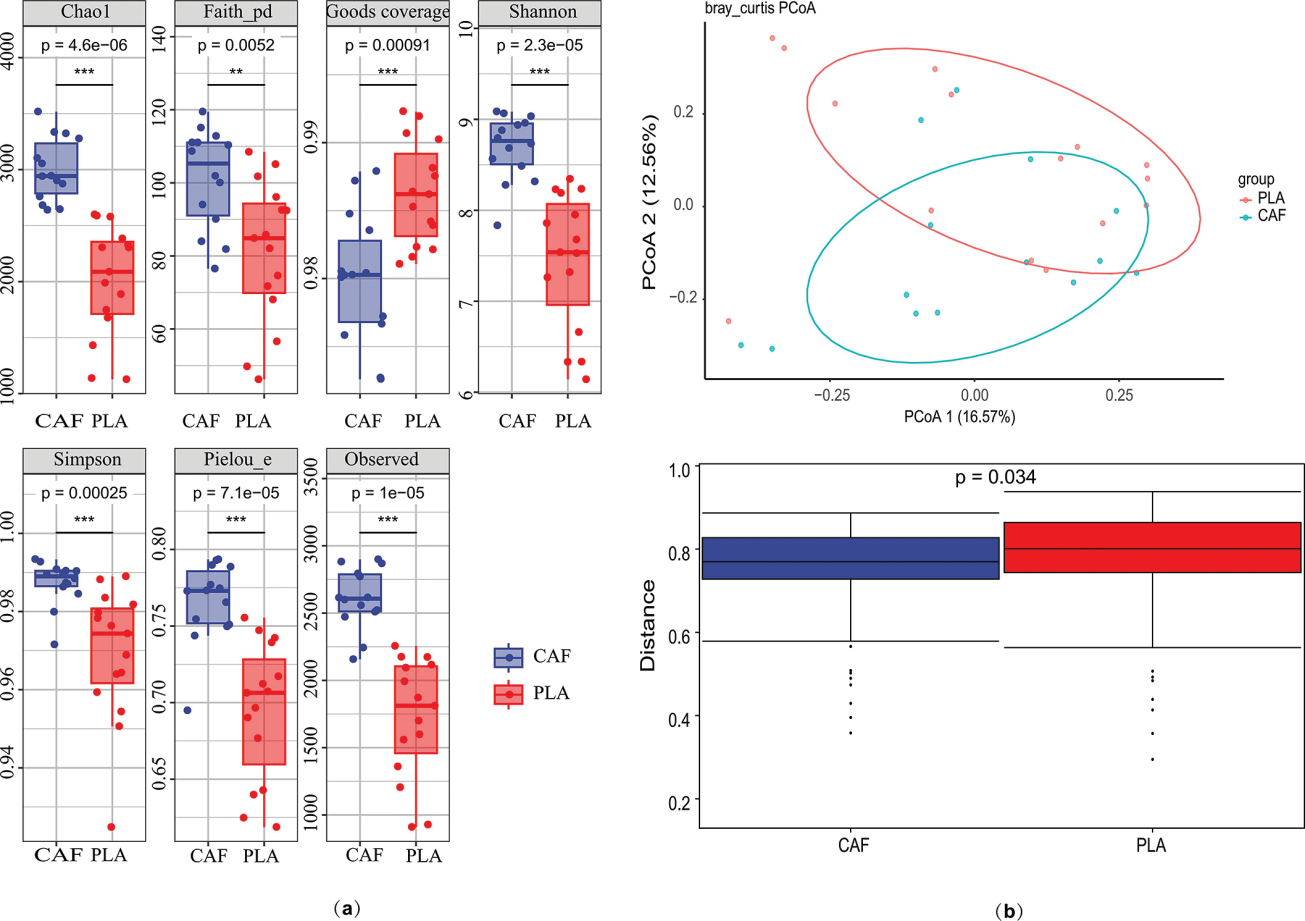
Gut microbiome analysis under caffeine supplementation. (a) Wilcoxon rank-sum tests were used to compare alpha diversity estimates of bacterial communities (p < 0.05). (b) Principal coordinate analysis (PCoA) based on Bray-Curtis distance at the ASV level and inter-group difference analysis were performed to compare data between groups, which were divided into experimental (CAF) and control (PLA) groups. *p < 0.05.

Alpha diversity of the gut microbiota was assessed using Chao1, Shannon index, and Observed Species metrics. All indices consistently demonstrated significantly higher bacterial diversity in the experimental (treat) group compared to the control group (p < 0.05 for all metrics; Figure 4a).Beta diversity analysis based on Bray-Curtis distances revealed significant compositional differences between groups at the ASV level (p = 0.001, PERMANOVA). Principal coordinate analysis (PCoA) explained 29.13% of the total variance in bacterial communities under caffeine supplementation. The first two principal coordinates (PCoA1 and PCoA2) accounted for 16.57% and 12.56% of the variance, respectively. Distinct clustering patterns were observed, with 95% confidence ellipses confirming separation between the control and experimental groups. Inter-group differential analysis further confirmed significant differences (p = 0.034, Figure 4b).

**FIG. 5:**
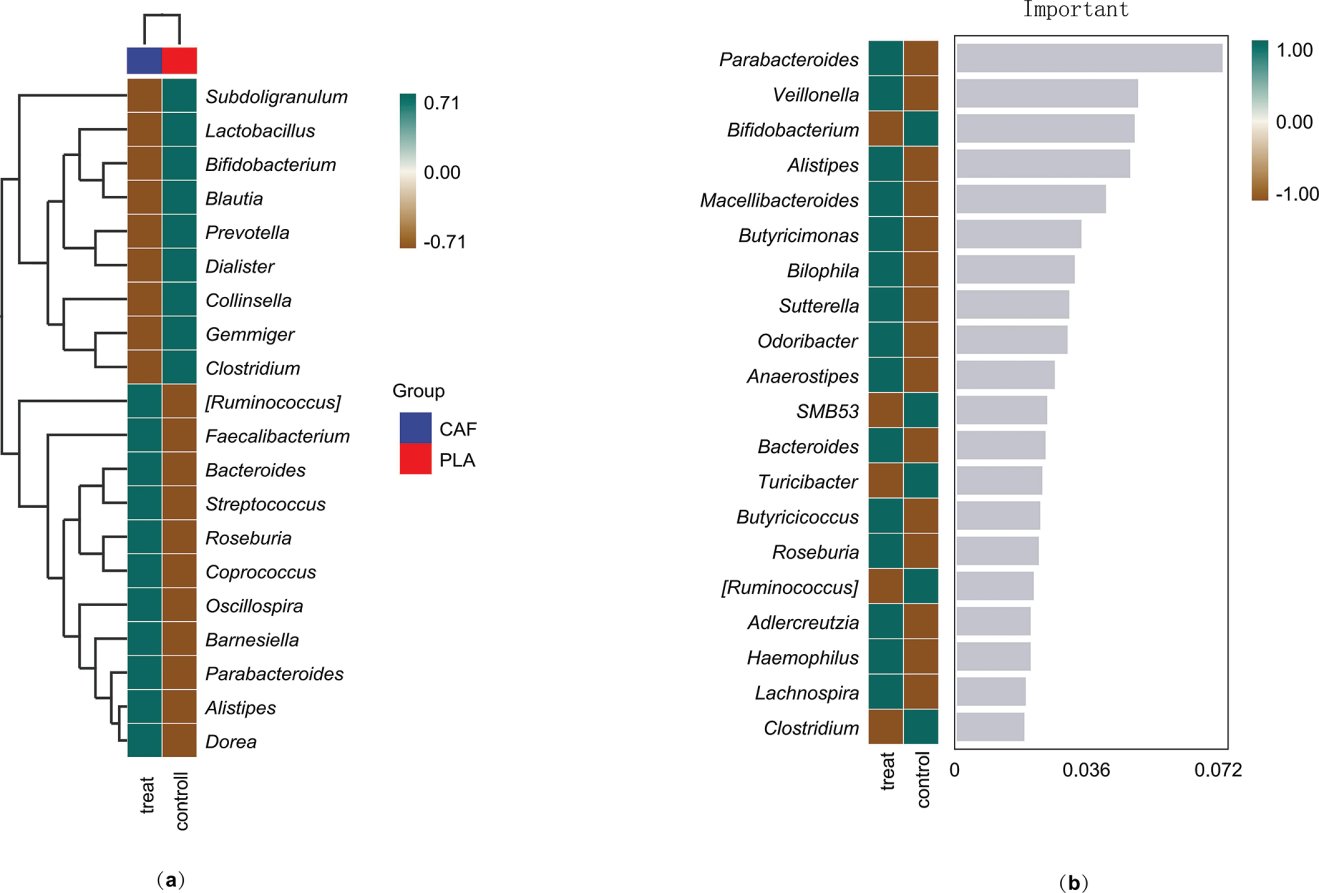
Two approaches were used to analyze taxonomic biomarkers of gut microbiota related to caffeine. (a) A heatmap visualized the relative abundance of the top 20 biomarkers linked to intensity. (b) The random forest method identified and ranked the top 20 relevant genera by their contribution.

To explore the differences in microbial community composition (β-diversity) mainly attributed to species distribution, two methods were used. First, heatmaps compared species composition changes among groups and analyzed the distribution trends of species abundance within groups, based on the abundance data of the top 20 genera with the highest average abundance (Figure 5a). Second, the random forest algorithm identified potential taxonomic biomarkers and classified microbial community samples effectively, robustly, and accurately. At the genus level, ten-fold cross – validation was done to get the species importance scores of the classifier model. Species importance to the model decreases from top to bottom; highly important species can be seen as marker species for group differences. *Parabacteroides, Veillonella*, and *Bifidobacterium* have relatively high importance scores of 0.07, 0.049, and 0.048, respectively (Figure 5b).

### Analysis based on structural equation modeling of the regulation of athletic performance in football players by caffeine through gut microbiota modulation

The structural equation model demonstrated excellent fit to the data, with all indices meeting established thresholds for good model fit: Comparative Fit Index (CFI) = 0.9, Tucker-Lewis Index (TLI) = 0.9, Root Mean Square Error of Approximation (RMSEA) = 0.06 (90% CI: 0.03–0.08), and Standardized Root Mean Residual (SRMR) = 0.04. The measurement models exhibited strong internal consistency, with Cronbach’s α values of 0.8 for the gut microbiota diversity latent factor (Chao1, Shannon, Observed_species) and 0.9 for the athletic performance improvement composite (Δ agilityshooting, Δ T-test, Δ 30-m sprint).

Caffeine supplementation significantly increased gut microbiota diversity (β = 0.5, p < 0.001, 95% CI [0.3, 0.6]), which in turn predicted greater improvements in athletic performance (β = 0.3, p = 0.007, CI [0.1, 0.6]). However, no direct effect of caffeine on performance was observed (β = 0.2, p = 0.2, CI [-0.09, 0.5]). Mediation analysis revealed that 33.3% of caffeine’s total effect on performance was mediated by microbiota diversity (indirect effect: β = 0.2, p = 0.01, CI [0.04, 0.3], Table 2).

**TABLE 2:** Direct and Indirect Effects.

Pathway	β (SE)	95% CI	p-value
CAF → Gut Microbiota	0.5 (0.08)	[0.3, 0.6]	< 0.001***
Gut Microbiota → Performance	0.3 (0.12)	[0.1, 0.6]	0.007**
CAF → Performance (Direct)	0.2 (0.1)	[-0.09, 0.5]	0.2

Sensitivity analyses confirmed the robustness of these findings. Adjusting for baseline Yo-Yo test scores did not attenuate the mediation effect (β = 0.1, p = 0.03). A fully mediated model excluding the direct caffeine-to-performance pathway showed inferior fit (ΔCFI = 0.06, p < 0.05), supporting partial mediation. Multivariate normality assumptions were met (Mardia’s test, p > 0.1), and no influential outliers were detected (Cook’s distance < 0.1 for all observations). These results underscore the critical role of gut microbiota in translating caffeine’s ergogenic effects into measurable athletic performance gains.

## DISCUSSION

The present study provides novel evidence that caffeine supplementation enhances the athletic performance of football players, with gut microbiota diversity playing a significant mediating role. Our findings align with existing literature on caffeine’s ergogenic effects but extend this understanding by elucidating a potential mechanistic pathway involving microbial modulation. The observed improvements in agility, sprint performance, and technical execution in the caffeine group corroborate previous reports that moderate caffeine intake (3.0–6.0 mg/kg BM) enhances neuromuscular recruitment and anaerobic capacity [5, 7]. However, the absence of a direct caffeineperformance effect in our structural equation model suggests that traditional explanations, such as adenosine receptor antagonism [9], may not fully account for caffeine’s benefits in football-specific tasks. Instead, our mediation analysis highlights gut microbiota diversity as a critical intermediary, explaining 33.3% of caffeine’s total effect on performance. This underscores the necessity of integrating microbiome science into sports nutrition research.

The gut microbiota alterations observed in the caffeine group— marked by increased alpha diversity and enrichment of genera like *Prevotella* and *Bacteroides*—resonate with prior studies linking these taxa to enhanced carbohydrate metabolism and anti-inflammatory responses [16, 17]. Such microbial shifts may optimize energy harvest and reduce exercise-induced oxidative stress, indirectly supporting high-intensity intermittent efforts characteristic of football [21]. Notably, the elevated abundance of *Veillonella*, a lactate-utilizing genus, aligns with evidence suggesting its role in improving endurance by recycling metabolic byproducts [19]. These findings collectively suggest that caffeine’s ergogenic properties may stem not only from direct physiological effects but also from fostering a microbiome environment conducive to athletic performance.

Notably, the elevated abundance of *Veillonella*, a lactate-utilizing genus, aligns with evidence suggesting its crucial role in improving endurance by recycling metabolic byproducts [19]. *Veillonella* species can convert exercise-induced lactate into propionate through the methylmalonyl-CoA pathway, potentially creating a beneficial metabolic cross-feeding relationship where exercise-generated lactate becomes a substrate for microbiome-derived Short-chain fatty acid (SCFAs) that may subsequently enhance oxidative capacity and reduce perceived exertion during high-intensity efforts. This microbial-mediated lactate shuttle could be particularly advantageous during the repeated sprint and technical execution tasks where rapid recovery between efforts is essential for maintaining performance.

Our results also intersect with emerging research on athlete microbiomes, which report higher microbial diversity and functional redundancy compared to sedentary individuals [18, 20]. The caffeineinduced microbial changes observed here share some similarities with patterns reported in athletes engaged in prolonged training. While our data suggest a potential relationship between caffeine supplementation and exercise-associated microbial profiles, we cannot conclusively state that caffeine mimics exercise-induced adaptations. This preliminary observation warrants further investigation, particularly regarding whether caffeine might interact with training to influence microbiome characteristics.

Recent systematic evidence has substantially expanded our understanding of the bidirectional relationship between physical exercise and gut microbiota composition. Santana Pereira et al. [45] demonstrated that regular physical activity across various intensities and modalities induces favorable alterations in gut microbiota composition and enhances intestinal integrity. Their systematic review revealed that these exercise-induced microbial adaptations appear dose-dependent, with higher training intensities correlating with more pronounced beneficial shifts in microbial communities. This relationship is particularly relevant to our findings, as the caffeine-induced microbial changes we observed parallel those reported in response to high-intensity training, suggesting potential synergistic mechanisms between caffeine supplementation and exercise adaptation pathways.

Furthermore, comparative analyses between elite soccer players and less active individuals have revealed distinct microbial signatures associated with athletic performance. Petri et al. [46] documented significantly greater abundances of specific beneficial microbial taxa in elite football players compared to moderately active or sedentary individuals. These athlete-specific microbiome profiles were characterized by enrichment of bacteria involved in short-chain fatty acid production and enhanced metabolic capacity for energy harvest from complex carbohydrates. The increased abundance of *Veillonella* observed in our caffeine group notably corresponds with the microbial signatures identified in elite athletes by Petri and colleagues, suggesting that caffeine supplementation may accelerate the development of a “performance-optimized” microbiome or enhance the metabolic functions of existing microbial communities.

The convergence of our findings with these recent studies suggests a potential “athletic microbiome” characterized by specific taxonomic and functional adaptations that support elite performance. Caffeine appears to modulate the gut ecosystem in ways that parallel exercise-induced adaptations, potentially offering a complementary intervention strategy to optimize the gut-muscle axis in athletes. This may be particularly valuable during intensive training blocks, competition periods, or rehabilitation phases when maintenance or enhancement of the performance-supporting microbiome is desired but training loads must be managed carefully.

Several limitations must be acknowledged. First, the sample size, though statistically adequate, was restricted to male national-level athletes, limiting generalizability to female or amateur populations. Second, the 7-day intervention period precludes conclusions about long-term microbial and performance adaptations. Third, while 16S rRNA sequencing identified taxonomic shifts, metagenomic or metabolomic analyses are needed to clarify functional pathways linking specific microbes to performance outcomes. Finally, the placebo (vitamin C) may not be physiologically inert, though its lack of effect on performance metrics mitigates this concern.

Future studies should explore dose-response relationships between caffeine, microbiota modulation, and performance, as higher doses might elicit stronger microbial or direct effects. Longitudinal designs tracking microbial dynamics across training seasons could reveal whether caffeine sustains microbiome benefits under physiological stress. Additionally, interventions combining caffeine with probiotics or prebiotics may further optimize gut-mediated ergogenic effects. Finally, investigating gender-specific responses and interactions between caffeine, diet, and genetics will enhance personalized nutrition strategies for athletes.

## CONCLUSIONS

This study provides compelling evidence that caffeine supplementation enhances football-specific athletic performance, with gut microbiota diversity acting as a significant mediator of these effects. The 7-day intervention demonstrated that caffeine intake (3 mg/kg body mass) significantly improved agility, sprint capacity, and technical execution in male national-level football players. Crucially, structural equation modeling revealed that 33.3% of caffeine’s ergogenic impact was attributable to increased gut microbial diversity, particularly through the enrichment of Prevotella, Bacteroides, and Veillonella— genera associated with enhanced metabolic efficiency and exercise recovery. These findings challenge traditional explanations of caffeine’s performance benefits by highlighting the microbiome as a novel mechanistic pathway.

The results underscore the importance of integrating gut microbiota analysis into sports nutrition research, offering a foundation for personalized interventions targeting microbiome optimization. Future studies should investigate long-term caffeine-microbiota interactions, gender-specific responses, and synergistic effects of caffeine with probiotics or dietary fiber. Such advancements could refine evidencebased strategies to maximize athletic performance while minimizing physiological strain. Ultimately, this work bridges the gap between nutritional ergogenics and microbiome science, paving the way for holistic approaches in sports medicine.

## Data Availability

The data presented in this study are original and were collected specifically for this research. They are not openly available in a public repository but are available from the corresponding author upon reasonable request and with the necessary considerations for participant confidentiality and ethical restrictions.
